# Asking patients if they have any questions can help improve patient satisfaction with medical team communication in the emergency department

**DOI:** 10.1186/s12873-024-01001-1

**Published:** 2024-05-20

**Authors:** Eleonora Dafne Frau, Dea Degabriel, Giorgia Luvini, Roberta Petrino, Laura Uccella

**Affiliations:** 1 Internal Medicine Department, Ospedale Regionale di Lugano -EOC - via Tesserete 46, Lugano, 6900 Switzerland; 2 Emergency Medicine Department, Ospedale Regionale di Lugano - EOC - via Tesserete 46, Lugano, 6900 Switzerland

**Keywords:** Emergency department, Communication, Intervention, Patient satisfaction, Patient experience

## Abstract

**Background:**

It is well known that patient satisfaction with medical communication in the emergency department (ED) improves patient experience. Investing in good communication practices is highly desirable in the emergency setting. In the literature, very few studies offer evidence of effective interventions to achieve this outcome. Aim of the study is to evaluate whether encouraging emergency physicians to ask if patients have questions at the end of the visit would improve patient satisfaction with medical communication.

**Methods:**

The physicians of two EDs in Lugano, Switzerland, were invited by various methods (mailing, newsletter, memo pens and posters, coloured bracelets etc.) to implement the new practice of asking patients if they had questions before the end of the visit. Patients discharged were consecutively enrolled. Participants completed the modified CAT-T questionnaire rating their satisfaction with medical communication from 1 (very poor) to 5 (excellent). Data such as age, means of arrival, seniority of the physician etc. were also collected. Statistical analysis was performed with Bayesian methodology. The results were compared with those of a similar study conducted one year earlier.

**Results:**

517 patients returned the questionnaire. Overall, patients’ satisfaction with communication in the ED was very good and improved from the previous year (percentage of fully satisfied patients: 68% vs. 57%). The result is statistically significant (C: I: 51.8 − 61.3% vs. 63.9 − 71.8% *p* = 0.000). Younger patients (< 30 ye22ars old) were slightly less satisfied. Waiting time did not affect perception of communication.

**Conclusion:**

This study implements a concrete way to improve patients’ satisfaction with medical communication in the ED. The intervention targeted only one item of the CAT-T (“Encouraged me to ask questions”) but it generated an overall perception of better communication from patients discharged from the ED. The study also confirms that there are some objective elements that can alter perception of quality of medical communication by patients (age, seniority of the physician), in agreement with the literature. In conclusion, focusing physicians’ attention on asking patients whether they have questions before discharge helps improving overall patient satisfaction with medical communication in the ED. This may lead to changes in physicians’ clinical practice.

**Supplementary Information:**

The online version contains supplementary material available at 10.1186/s12873-024-01001-1.

## Background

A more satisfied patient is a healthier one and is more likely to favourably interact with the healthcare system and healthcare providers. This is something that we now know from multiple studies [1–3]. Communication between physicians and patients promotes interactions and builds positive relationships [4]. There is therefore considerable interest in investing in practices which can enhance patient satisfaction [5].

Improving communication practices is not easy. Educational practices do not easily produce behavioural changes [6]. Many physicians intend to change their behaviour but they fail to successfully realise this goal. Knowledge and good intentions are insufficient by themselves to produce behavioural change [6].

In October 2021 an observational cross-sectional study attempting to assess patient satisfaction and identify objective elements affecting the quality of communication perceived by patients was conducted in the Emergency Department (ED) of two city hospitals sharing the same staff in the south of Switzerland [7]. The study, published in 2023, was carried on to address a need for more quantitative data in the field of communication, in particular in the emergency medicine setting. The ED setting is characterised by limited time to spend with the patient, a busy and often chaotic environment, physicians not being in a position to establish a durable relationship with the patient, patient’s fears and worries in the context of an emergency [8, 9]. This is why identifying quantitative aspects which are significantly correlated with patient satisfaction can aid the development of targeted interventions and ultimately improve patient health.

In the study performed in October 2021 patients were asked to complete the Italian CAT-T survey [9–11] and additional objective data was gathered by the treating physicians (age, means of arrival, time spent waiting, etc.). Participants were found to be generally satisfied with the quality of communication with the medical team. Patients’ responses were generally good (all averaging more than 4.4 on a scale from 1 to 5, where 1 meant very bad and 5 meant excellent). The results observed were comparable to other studies mentioning use of the same tool to assess patient satisfaction [9, 11]. Significant results of the study included younger patients (< 30 years old) expressing lower overall satisfaction, as well as patients brought to the hospital by ambulance. Patients who visited the larger, of the two hospitals were generally more satisfied. Long waiting times, surprisingly, did not show lower satisfaction. Of particular interest was the item with the lowest mean score, which pertained to patients not feeling encouraged by the medical team to ask questions during the doctor-patient interview [6]. This item was therefore identified as a priority and a viable first target for efforts to increase patient satisfaction.

This study aims to investigate whether implementing a specific intervention addressing this item by encouraging doctors to react in their interaction with patients could lead to improved patient satisfaction with communication in the ED.

## Methods

### Study design

This is an observational, prospective, cross-sectional study that represents an attempt to implement an intervention aimed at improving the perception of doctor-patient communication by encouraging ED physicians to ask the patient before the end of the visit if he or she had any questions. The decision was to implement only one intervention at a time to be able to measure its effects.

To encourage colleagues to ask the patient if they had more questions, several strategies were implemented before the study and some during the study.

1. Department meetings were held in which the results of the previous study were communicated, with emphasis on the relatively low score collected by question number 10 of the CAT-T questionnaire.
2. Before the start of the month of observation, a reminder email was sent about the results of the previous study.
3. Each physician on the ward received a pen with a question mark depicted on it, with the stated purpose of it serving as a memento.
4. Every day each doctor on the ward was given a coloured bracelet with a question mark for the same stated purpose.
5. In every patient’s cubicle and doctors’ office a poster with a question mark was hung.
6. The computer system by which patients’ discharge letters are generated reminded colleagues with a red script visible only to physicians to ask the patient if they had any questions.

The study was conducted from October 1–31, 2022 in the same two hospitals as the first study (one big hospital and one smaller hospital with the same medical, nursing and administrative staff, the first with 30,000 ED visits/year and the second with around 8000 patients/year in the ED). The 32 doctors (17 residents and 15 seniors) visiting the patients were the same as the previous year except 5 residents who had changed.

The bigger hospital offers comprehensive medical services including intensive care, surgery, invasive cardiology, interventional angiography, neurosurgery, and a stroke unit, covering a wide range of specialties. In contrast, Hospital B primarily focuses on outpatient care.

Additionally, the smaller hospital differs in terms of building infrastructure, with this being a newer construction. Geographically, the smaller hospital is situated in the city center, whereas the bigger is located more on the outskirts.

All individuals aged 18 or older who had been released from the Emergency Department were considered eligible for participation in the study, provided they did not meet any of the following exclusion criteria: critical illness, psychiatric disorders, cognitive impairment (such as dementia), and non-Italian language proficiency. Patients were enrolled in a consecutive manner without any selective criteria.

Following their discharge from the Emergency Department, patients were requested to complete the CAT-T questionnaire in its Italian version. The CAT-T is a validated tool designed to assess patients’ perceptions of physicians’ performance, specifically focusing on interpersonal and communication skills [9–12].

The CAT-T is a survey comprising 15 items that assess responses on a 5-point scale (1 = poor, 5 = excellent). The survey’s core consists of 14 items, concentrating on communication within the doctor–patient relationship. The additional item, number 15 (“the staff treated me with respect”), was excluded from the analysis as it did not align with the study’s focus.

The CAT-T has undergone psychometric validation to measure patient perceptions of communication with medical teams and has been previously applied in the emergency care setting [9–12]. Detailed information regarding scale development processes and psychometric properties can be found in the original CAT-T article [9].

Patients were asked to participate in the study and filled in the CAT-T while about to leave the ED, after a brief oral presentation of the study by the doctor who had treated them. The questionnaire was anonymous.

At the same time a medical annex was filled out by physicians, who recorded some data such as gender, age, medical category (surgical or medical), function held by the physician who examined the patient (junior, senior or both), the means of arrival (ambulance, referral from the attending physician or patient initiative), the triage code with presenting complaint, the waiting time before the visit and the overall length of stay in the ED, the time of day and whether the day was a weekday or weekend (or holiday).

The additional data collected were not connected with patient or physician names and all patients provided verbal consent before study enrolment.

The questionnaire was completed at the end of the emergency room evaluation, in the absence of the doctor who had assessed the patient. The patient then submitted the completed questionnaire into a box at the exit. Also the accompanying medical form was anonymous. The questionnaire and the medical annex were linked by a code and deposited by the physician in the same box, so after depositing the questionnaire and form in the box, the patient could not be identified.

Nine months after the study was completed, the emergency physicians involved in the study were asked to rate the following questions on a scale from 1 (not at all agree) to 5 (completely agree):

- How much did you adhere to the request of asking the patients if they had more questions?
- How much has this question now become part of your routine clinical behaviour?

### Outcome measures

The primary outcome of the study was assessing if there was a variation in patients’ satisfaction with item 10 of the CAT-T (“Encouraged me to ask questions”).

Secondary outcome of the study was assessing if there was a variation in any other item of the CAT-T.

The regional Ethics Committee (Cantonal Ethics Committee of Ticino) approved this research project without requiring formal written consent because consent was implied by completion of the questionnaire.

### Statistical analysis

Statistical analysis was carried out using the open source Python packages “Pandas”, “NumPy”, “SciPy”, “Seaborn” and “PyMC” for Mac Os X versions 1.4.1, 1.21.2, 1.7.3, 0.11.2 and 3.11.14, respectively. Statistical significance was considered fulfilled based on highly credible intervals of parameter estimates. Confidence Intervals (CI) were calculated at 95%. For better clarification, we also calculated the relative risk, defined as the ratio of the risks for an event for the exposure group (being totally satisfied) to the risks for the non-exposure group (being somewhat unsatisfied).

We used a Bayesian approach (albeit with uninformative a-priori) that does not have the sample size limitations that frequentist methods relying on asymptotics have. Bayesian approach addresses uncertainty by producing “wider” Confidence Intervals: when the data are many the CI is narrow, whereas if the data are few the range widens. If the CIs of two different subgroups are separated (e.g., young vs. old), the data has strong statistical significance [13].

We calculated a P value only as a usual reference.

The scores of the questionnaire (assigning a numerical score to a qualitative judgment such as “excellent” or “poor”) are ordinal but not metric measures: this poses a problem, because averaging the values obtained in this way is meaningless: these variables are neither quantitative nor metric. Therefore we decided, for each item of the questionnaire, to divide the scores into “satisfied” and “non-satisfied” patients. Only scores of “5” (excellent) were considered a fully satisfied answer, and all the answers below 5 were considered non fully satisfied. We then calculated the proportion of people who gave a “fully satisfied” answer in the questionnaire and compared it with the proportion of people who gave a “fully satisfied” answer in the previous study conducted in 2021. This allowed us to see if there had been an impact from the intervention carried out.

## Results

During the study period (October 1–31, 2022), a total of 1896 patients were discharged from our ED (1497 from Hospital A and 399 from Hospital B).

A total of 553 patients were enrolled in the study. Twelve patients were excluded from the initial analysis because they lacked demographic and operational data (age, gender, medical category, wait time, etc.). Another 24 patients were subsequently excluded because they did not return the questionnaire, so statistics were performed on a sample of 517 patients.

All patients who were invited completed the questionnaire, except for 24 patients who did not return it. Other patients (1443 subjects) were not included in the study primarily because physicians lacked sufficient time to invite them.

The characteristics of patients who responded to the questionnaire did not differ significantly in age, gender or type of presented complaint, from the population of all patients discharged from ED during the examined period. A single imbalance was observed with respect to surgical vs. medical patients with a greater proportion of surgical patients in comparison with the previous year.

Characteristics of the final sample are shown in Table 1. The complete data-sheet is available in Online Resource 1.

**Table 1:** Characteristics of the final sample

Characteristic	Nr of pts 2022 (517)	% 2022	Nr of pts 2021 (394)	% 2021
**Sex**				
M	272	52.7	201	51
F	245	47.3	193	49
**Age**				
≤ 30y	118	22.8	89	22.6
> 30–65 y/o	290	56	218	55.3
> 65–80 y/o	75	14.5	65	16.5
> 80 y/o	34	6.5	22	5.6
**Medical category**				
Surgery	253	48.9	230	58.4
Medicine	264	51.2	164	41.6
**Day**				
Working day	392	75.8	284	72.1
Holiday/week-end	125	24.2	110	27.9
**Time spent in the ED**				
< 1 h	132	25.3	110	27.9
1–3 h	258	49.9	190	48.2
3 > time < 6 h	108	20.8	84	21.3
> 6 h	14	2.7	10	2.5
**Time spent waiting**				
< 30 min	368	71.7	271	68.8
30 > waiting time < 60 min	75	14.5	60	15.2
> 60 min	71	14	63	16
**Time spent in ED bay**				
**Means of conveyance to the ED**				
Announced by family physician	40	7.7	19	4.8
Ambulance	24	4.6	25	6.4
By own initiative	453	87.6	350	88.8
**Hospital site**				
Hospital A	374	72	251	63.7
Hospital B	143	27.6	143	36.3
**Physician function**				
Assistant in training	220	42	203	51.5
Senior	165	31	97	24.6
Assistant in training AND senior	132	25	94	23.9

Scores obtained indicated that overall, patients were satisfied with doctor–patient communication. All items received a mean score over 4.5 on a scale from 1 to 5. The proportion of fully satisfied patients (patients who assigned a score 5) ranged from 68 to 85%. Mean scores, standard deviations and proportion of completely satisfied patients are shown in Table 2.

**Table 2:** Mean score, standard deviation and proportion of completely satisfied patients for each of the comparison years

Questions	Mean 2022	Mean 2021	St. Dev. 2022	St. Dev. 2021	Score = 5 (%) 2022	Score = 5 (%) 2021
1. Greeted me in a way that made me feel comfortable	4.71	4.74	0.64	0.54	79	73
2. Treated me with respect	4.82	4.81	0.47	0.50	85	80
3. Showed interest in my ideas about my health	4.74	4.74	0.54	0.56	78	74
4. Understood my main health concerns	4.74	4.71	0.56	0.59	78	71
5. Paid attention to me (looked at me, listened carefully)	4.76	4.77	0.57	0.50	81	75
6. Let me talk without interruptions	4.78	4.81	0.53	0.49	79	79
7. Gave me as much information as I wanted	4.75	4.76	0.54	0.53	81	74
8. Talked in terms I could understand	4.74	4.79	0.49	0.47	78	77
9. Checked to be sure I understood everything	4.72	4.72	0.60	0.55	78	71
10. Encouraged me to ask questions	4.55	4.46	0.77	0.82	68	57
11. Involved me in decisions as much as I wanted	4.60	4.57	0.72	0.72	70	63
12. Discussed next steps, including any follow-up plans	4.70	4.64	0.60	0.65	73	65
13. Showed care and concern	4.71	4.68	0.60	0.63	78	70
14. Spent the right amount of time with me	4.65	4.66	0.71	0.57	76	70

The item that received the highest score was “Treated me with respect” (mean 4.82), whereas the lowest score was attributed to the item “Encouraged me to ask questions” (mean 4.55).

The percentage of fully *satisfied* patients, who responded with 5 to individual items, increased in 13/14 items from 4 to 11%, while in one (number 6), it remained unchanged compared to the previous study. In particular, the largest increase occurred in item number 10, “Encouraged me to ask questions.”

Comparing the proportion of patients who gave “satisfied” answers in the present study with the proportion of patients who gave “satisfied” answers in 2021 in items number 1, 2, 4, 5, 7, 9, 10, 11, 12, 13 and 14 we found a statistically significant difference with separated CIs in favour of a better perception of medical communication by patients in 2022. Detailed CIs and p values are shown in Table 3.

**Table 3:** Comparison of proportion of fully satisfied (score = 5) answers between 2021 and 2022, C.I.s, relative risk and p value

Item: The doctor(s)….	C.I. 2021 (% of satisfied answers)	C.I. 2022 (% of satisfied answers)	Odds Ratio (95%C.I.)	*p* value
1. Greeted me in a way that made me feel comfortable	67.4 -76%	74.7 − 81.8%	1.42 (1.04–1.92)	0.021*
2. Treeted me with respect	74.4 − 82.4%	81.2 − 87.6%	1.63 (1.16–2.28)	0.017*
3. Showed interest in my ideas about my health	68.6 − 77.3%	74.0 − 81.1%	1.25 (0.92–1.69)	0.110
4. Understood my main health concerns	65.5 − 74.7%	78.9 − 81.2%	1.43 (1.06–1.94)	0.009*
5.Paid attention to me (looked at me, listened carefully)	69.2% − 77.(%	77.5 − 84.4%	1.43 (1.04–1.96)	0.006*
6. Let me talk without interruptions	73.4 − 81.4%	78.6 − 85.3%	0.99 (0.72-1-37)	0.087
7. Gave me as much information as I wanted	73.9 − 76.6%	75.2 − 82.4%	1.51 (1.10–2.07)	0.023*
8. Talked in terms I could understand	71.2 − 79.6%	77.0 − 83.8%	1.06 (0.77–1.45)	0.068
9. Checked to be sure I understood everything	65.2 − 74.2%	73.8 − 81.2%	1.43 (1.06–1.94)	0.007*
10. Encouraged me to ask questions	51.8 − 61.3%	63.9 − 71.8%	1.61 (1.23–2.12)	0.000*
11. Involved me in decisions as much as I wanted	57.2 − 66.7%	66.0 − 73.8%	1.21 (0.92–1.59)	0.011*
12. Discussed next steps, including any follow-up plans	58.6 − 68.3%	69.1 − 76.9%	1.45 (1.10–1.91)	0.002*
13. Showed care and concern	64.6 − 73.4%	74.1 − 81.3%	1.51 (1.12–2.03)	0.003*
14. Spent the right amount of time with me	63.5 − 72.8%	71.0 − 78.3%	1.36 (1.01–1.82)	0.033*

With regard to primary outcome, patients’ satisfaction with Q10 increased significantly (OR 1.61, C.I. 1.23–2.12, *p* < 0.000).

**Fig. 1:**
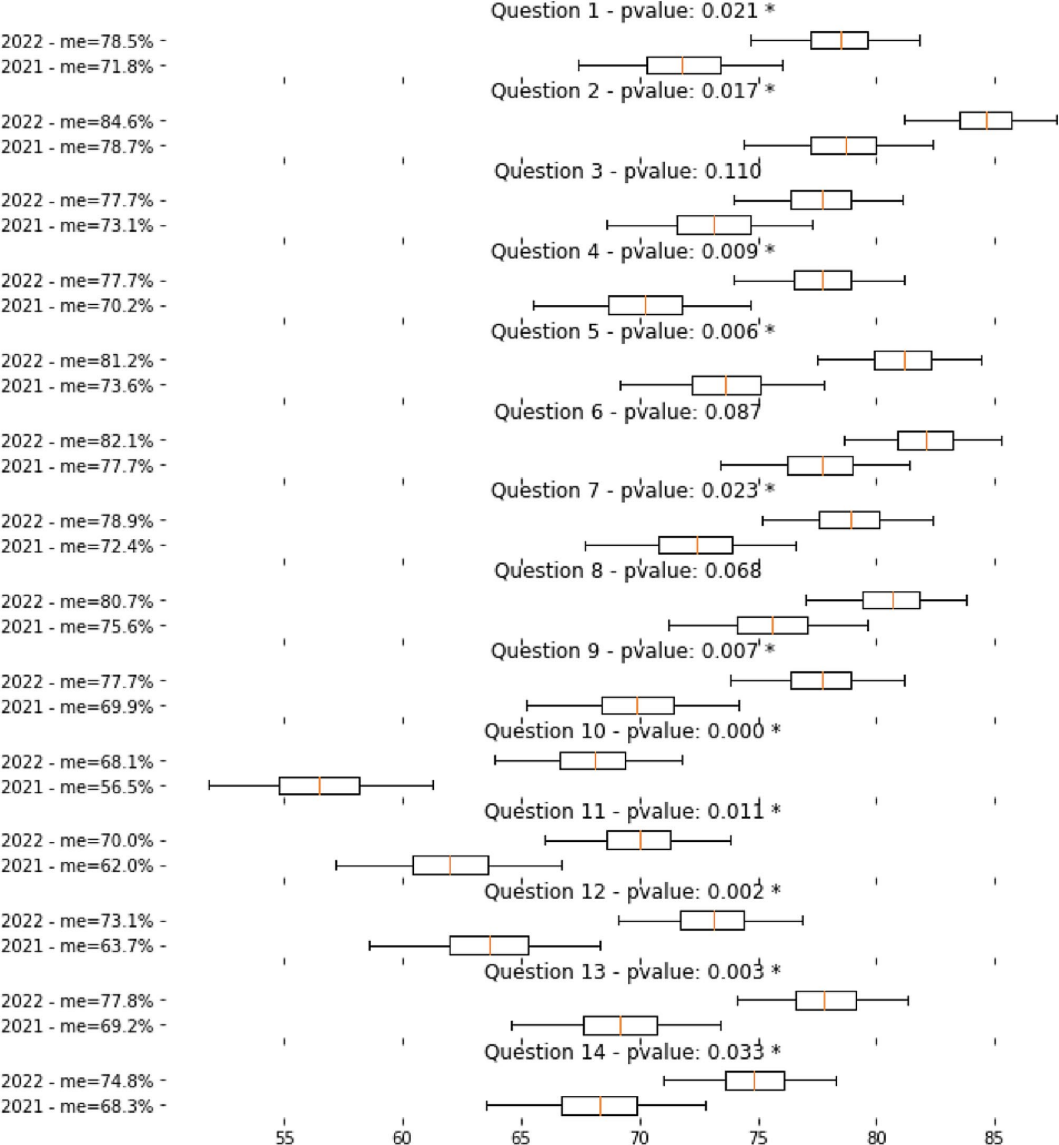
comparison of satisfied answers between 2021 and 2022. Cis for completely satisfied responses (score = 5) are displayed and compared for 2021 and 2022 (after the implemented intervention)

Figure 1 (comparison of satisfied answers between 2021 and 2022) visualises the differences.

Patients assessed by senior doctors tended to give more “satisfied” answers with respect to patients evaluated by residents, but the difference did not reach statistical difference.

Again, patients evaluated by both seniors and juniors were less “satisfied” than patients evaluated by only one physician. This difference was statistically significant (CI 22.1%-37.4% vs 14.4–22.1%, relative risk 161.8%; p 0.007).

Young patients under 30 years of age tended to be less “satisfied” than their older counterparts. This difference reached statistical significance with item 10, “Encouraged me to ask questions”, (percentage of unsatisfied answers CI 40.4 − 60.8% vs. 17.0 − 38%, relative risk 187%; p 0.013).

In our study, the long waiting times, length of stay in the ED, surgical vs non surgical reason for consulting and weekend days did not generate less “satisfied” responses.

In the present study, as in 2021, patients transported to the ED by ambulance tended to be less “satisfied than patients referred by family doctor or self-referred. The difference did not reach statistical significance.

Nine months after the study completion, the physicians involved in the study were asked to rate on a scale from 1 (not at all agree) to 5 (completely agree) these two questions:

- How much did you adhere to the request of asking the patients if they had more questions?
- How much is this question now become part of your routine clinical behaviour?

The answers are detailed in Table 4.

**Table 4:** Answers of ED physicians after 9 months from the study (2022)

		1 = not at all (%)	2	3	4	5 = completely
How much did you adhere to the request of asking the patients if they had more questions?	Senior	1 (7%)	0 (0%)	3 (21%)	8 (57%)	2 (14%)
	Junior	0 (0%)	1 (5%)	3 (16%)	10 (55%)	4 (22%)
How much has this question now become part of your clinical practice?	Senior	0 (0%)	0 (0%)	0 (0%)	6 (42%)	8 (57%)
	Junior	2 (11%)	0 (0%)	2 (11%)	4 (22%)	10 (55%)

In the clinical practice of physicians after the study, a general perceived integration of the habit of asking the patient if he or she had further questions was observed more than during the study itself.

## Discussion

This study aimed at investigating whether implementing a specific intervention addressing item 10 by encouraging doctors to ask patients if they have any questions could lead to improved patient satisfaction with this question when answering the CAT-T questionnaire.

It seems that the implementation of behavioural change by physicians has worked and led to desired results.

This interventions offers a concrete way to improve patient satisfaction with medical communication in the ED.

The improvement promises to be persistent over time as the attitude of asking patients if they have questions seems to be acquired in the clinical practice of the ED physicians who participated in the study.

We received 517 responses out of 1896 patients discharged during the study period: patients were not included in the study primarily because physicians lacked sufficient time to invite them.

This may have created a selection bias by colleagues who might have chosen the more satisfied patients.

The patient demographics were largely similar compared to the previous year, with the difference that in 2022 more questionnaires were completed, likely due to increased attention from colleagues.

Therefore, even if there were indeed a selection bias, the fact that more patients responded in the second year strengthens our results. The only imbalance we observed was that in 2022 there were more surgical patients than the previous year. We did not find any similar results in the literature and the statistical analysis did not show any differences between surgical and non surgical group.

The intervention carried out targeted only one item (“Encouraged me to ask questions”) but it generated an overall perception of better communication from patients discharged from the ED.

It is an interesting finding that item 10, though improved, still achieves the lowest value even after the intervention. The literature extensively documents clinicians’ perceived failure to encourage patient questions, a concern highlighted prominently in studies using CAT-T [10, 11]. The reasons for this are likely complex and cannot be fully explained by survey data alone. While improving training in consultation closure is important, additional barriers such as time pressure and reluctance to engage in lengthy discussions during busy schedules may contribute. Similarly, the findings suggest a need to foster greater patient involvement in care and decision-making [14].

There are many studies in the literature that examine medical communication in the ED [15–17] and address the need for improvements, but only a few offer evidence of actual changes that gave measurable improvements [18–20].

The society for Academic Emergency Medicine in 2011, during the consensus conference “Interventions to assure quality in the ED” stated that patient-centred care can be operationally measured in three domains: patient satisfaction, patient involvement and care related to patient needs [21]. Medical communication is one way of improving patient satisfaction and the conference stated that, although there is a lack of literature describing interventions to improve communication, such interventions should be identified and prioritised.

This study also confirms that there are some objective elements that can alter perception of quality of medical communication by patients (age, seniority of the physician), in agreement with the literature [7, 22].

In particular, the seniority of the physician tends to be directly proportional to patient satisfaction with communication, and this is in agreement with most recent studies. The reason why patients were less satisfied when managed by both senior and junior doctors, however, is not clear and we can only make some guesses.

The redundancy of questions asked by two different physicians, the possible perception of less attention by physicians and the partial transfer of communication responsibility could be possible explanations. Dugdale et al. argue that the delegation of certain tasks may reduce the time patients spend with their physicians [23].

In the present analysis, waiting times and day of the week do not seem to influence this same perception. This is only in agreement with part of the literature, but it cannot be ruled out that much depends on the context and country in which physicians operate and on many other factors that were not the subject of this paper [24].

Our study also confirms that young individuals are generally less satisfied with communication with the emergency room medical team, particularly regarding item 10. This might be attributed to younger individuals being more attuned to various aspects of information flow and communication, given their proficiency with technology and global connection. Additionally, they may harbor higher expectations regarding their ED treatment. Exposure to diverse media or contemporary culture possibly shapes these expectations, leading them to arrive at the ED with very specific expectations [7, 25].

### Limitations

This study also has some limitations.

This was a single-center study: further multi-center studies are needed to see if the results are generalisable. There might have been a potential Hawthorne effect because physicians were aware that a study on communication was being conducted. Furthermore, we decided to focus on discharged patients and our result do not cover inpatients. In the end, we do not know whether the improvement in communication and the changes in clinicians’ practice will last beyond years.

## Conclusions

In conclusion, focusing physicians’ attention on asking patients whether they have any questions shortly before discharge can help improve overall patient satisfaction with medical communication in the ED and lead to lasting changes in physicians’ clinical practice.

### Electronic supplementary material

Below is the link to the electronic supplementary material.

Supplementary Material 1

## Data Availability

The datasets generated during and/or analyzed during the current study are available in the supplementary material.

## Abbreviations

ED: Emergency department
CI: Confidence interval
